# Multistage designs for phase II clinical trials: statistical issues in cancer research.

**DOI:** 10.1038/bjc.1996.537

**Published:** 1996-10

**Authors:** A. Kramar, D. Potvin, C. Hill

**Affiliations:** Département de biostatistique et d'épidémiologie, Institut Gustave Roussy, Villejuif, France.

## Abstract

The main objective of phase II clinical trials is to estimate treatment efficacy on a relatively small number of patients in order to decide whether the treatment ought to be studied in large-scale comparative trials. They play a key role in the drug development process, since the results determine whether or not to proceed to phase III trials. Multistage designs for phase II clinical trials proposed by Gehan, Fleming, Simon and Ensign are described and compared. Gehan's and Simon's designs have two stages, Fleming's designs can have two or more stages, and Ensign's three-stage design combines the first stage of Gehan with the two stages of Simon. Phase II clinical trial protocols and reports should include a description of the design selected with a justification for the particular choice. The present practice is very far from this ideal.


British Journal of Cancer (1996) 74, 1317-1320
© 1996 Stockton Press All rights reserved 0007-0920/96 $12.00

### Multistage designs for phase II clinical trials: statistical issues in cancer research

A Kramar, D Potvin* and C Hill

*Département de biostatistique et d'épidémiologie et INSERM U351, Institut Gustave Roussy, 94805 Villejuif Cedex, France.*

**Summary** The main objective of phase II clinical trials is to estimate treatment efficacy on a relatively small number of patients in order to decide whether the treatment ought to be studied in large-scale comparative trials. They play a key role in the drug development process, since the results determine whether or not to proceed to phase III trials. Multistage designs for phase II clinical trials proposed by Gehan, Fleming, Simon and Ensign are described and compared. Gehan's and Simon's designs have two stages, Fleming's designs can have two or more stages, and Ensign's three-stage design combines the first stage of Gehan with the two stages of Simon. Phase II clinical trial protocols and reports should include a description of the design selected with a justification for the particular choice. The present practice is very far from this ideal.

**Keywords:** phase II clinical trial; multistage design

Testing a new treatment on humans requires care, time and resources, and decisions concerning the future of the new treatment are made at the end of each phase I, II or III of the drug development process. A treatment can be abandoned at any stage of its development. The objective of a phase I trial is to determine a maximum tolerated dose, safe for a phase II study of therapeutic activity. Toxicity is therefore the main end point in a phase I trial. The phase II evaluation of a new anti-cancer drug is a screen to determine whether the drug has anti-tumour activity worthy of future clinical evaluation. The main end point is tumour response, completed by monitoring of toxicity data. A typical phase II study includes less than 50 patients, the objective being to identify an active treatment or to reject an inactive treatment as soon as possible. Phase II clinical trials play a key role in the development of a treatment because the results determine whether or not to proceed with phase III trials. Phase III randomised trials study the efficacy of a new treatment in the clinical context, and the end points are generally survival and disease-free survival. They include a much larger number of patients than phase II studies, from a few hundred in rare diseases to several thousand if not ten thousand in more frequent cancer sites. One must therefore be sufficiently convinced of the activity of a treatment to decide whether or not it should be tested in a phase III randomised trial.

To determine the number of patients required for a phase II trial, different multistage designs have been developed. The first design was proposed by Gehan in 1961 and is still being widely used (Gehan, 1961), although rarely cited. It is a two-stage design which allows for the rapid rejection of an ineffective treatment at the end of the first stage, and provides an estimation of the success rate with a given precision, at the end of the second stage. Fleming (1982) developed multistage designs which enable early termination of a trial when the treatment is either clearly effective or clearly ineffective. Simon (1989) improved Fleming's two-stage design by minimising either the average or the maximum number of patients required under the hypothesis of treatment ineffectiveness. Recently, Ensign *et al.* (1994) developed a three-stage design which integrates Gehan's and Simon's designs. Each of these designs is subject to different constraints and the aim of this paper is to present the designs and discuss their advantages and disadvantages.

Correspondence: A Kramar
*Present address: Phoenix International Life Sciences Inc., 4625, Dobrin Street, Saint Laurent, Montréal, Québec, Canada H4R 2P7
Received 27 March 1996; revised 2 May 1996; accepted 9 May 1996

### General prinicples

The response to a treatment will be summarised as being a success or a failure. In oncology, a success is usually defined as either a complete response or as an objective response which also includes partial responses.

The therapeutic efficacy is evaluated by the parameter $\pi$, the true proportion of successes among a given population. If this true proportion of successes is less than or equal to a predetermined value $p_0$, which we shall call the maximal rate of inefficacy, the efficacy of the treatment will be considered as insufficient. If the true proportion of successes is greater than or equal to a predetermined value $p_1$, which we shall call the minimal rate of efficacy, the treatment will be considered as being sufficiently effective for further study in phase III trials.

Most statistical methods developed for the inclusion of patients in phase II trials are built in at least two stages. In the simplest case, $n_1$ patients are included in the first stage and $r_1$ successes are observed. Depending on the value of $r_1$, either the trial is stopped or recruitment continues by accruing a further $n_2$ patients into a second stage, among whom $r_2$ successes are observed. The cumulative number of successes is then $R_2 = r_1 + r_2$ out of $n_1 + n_2$ patients.

At each stage $k$, a decision for stopping the trial or continuing to accrue patients is taken depending on the observed cumulative number of successes $R_k$. The general procedure at each stage $k$ is as follows:

- If the total number of successes $R_k$ is less than a predetermined value, which we shall call the lower cut-off point of decision making, then the treatment is not considered to be sufficiently effective. The hypothesis of efficacy is then rejected. The error risk $\beta_k$ associated with this decision (type II error) is the probability of rejecting efficacy at the end of stage $k$ when in fact the treatment leads to a success rate at least equal to $p_1$
- If the total number of successes $R_k$ is greater than or equal to a predetermined value, which we shall call the upper cut-off point of decision making, then the treatment is considered sufficiently effective for further study in phase III trial. The hypothesis of inefficacy is thus rejected. The error risk $\alpha_k$ associated with this decision (type I error) is the probability of concluding in favour of efficacy at the end of stage $k$ when, in fact, the treatment is ineffective, i.e. when the success rate is $p_0$ or less.
- If the total number of successes is between the lower and the upper cut-off points, the trial continues and proceeds to the next stage by including more patients.

At the end of the last stage, there is a single cut-off point. If the total number of successes is at least equal to this cut-off point, one concludes in favour of efficacy, if the total number of successes is below the cut-off point, one concludes the ineffectiveness of the treatment.

The error risks $\alpha_k$ and $\beta_k$ associated with a wrong conclusion at each stage for each hypothesis are computed from binomial distributions, as are the overall error rates $\alpha$ and $\beta$.

### Different designs

### *Gehan's design*

Gehan (1961) proposed a design which rejects an ineffective treatment early when no success is observed among the first $n_1$ patients. Here $n_1$ is obtained from the following equation:

$$\beta_1 = \text{Probability (0 successes among } n_1 \text{ patients given } p_1) = (1-p_1)^{n_1}$$

This equation states that the probability of rejecting a treatment which has a true proportion of successes equal to $p_1$, after $n_1$ consecutive failures, is equal to $\beta_1$. For example, if $\beta_1 \approx 0.05$ and $p_1 = 0.30$, we find $n_1 = 9$ patients, since the probability of observing nine consecutive failures, if the true percentage of success is 30%, equals $(1-0.30)^9 = 0.040$ which is slightly less than 0.05.

If no successes are observed at the first stage, the trial stops and the treatment is considered ineffective with a $\beta_1$ error rate of 4%. A conclusion of efficacy, however, cannot be reached at the end of this first stage, no matter how many successes are observed, and therefore the risk $\alpha_1$ is taken to be 0.

If there is at least one success, the trial proceeds to the second stage. The goal of this second stage is to estimate the true proportion of successes $\pi$ with a given precision. The precision is defined as the estimated standard error of $\pi$. Tables giving the sample sizes for each stage are provided in Gehan's paper for several values of $p_1$, for $\beta_1 = 5\%$ or 10% and for precisions of 5% and 10%. These tables are also provided in a recent book (Machin *et al.*, 1996). The size of the second stage varies greatly, varying for instance, after the inclusion of nine patients, between 71 and 91 depending on the number of successes observed during the first stage, for a precision of 5%. For $p_1 = 0.20$ and $\beta_1 = 5\%$, which were the values used by Gehan in his original paper, one gets $n_1 = 14$. This particular result, $n_1 = 14$, is often quoted without any mention of the underlying hypothesis, but with reference to Gehan's paper.

Gehan's design is different from the designs presented below because it considers the sample size problem from an estimation point of view, while controlling the risk $\beta$ of rejecting an effective treatment. There is no need to specify a value $p_0$ of minimal efficacy, which would be used for controlling the probability $\alpha$ of accepting an ineffective treatment. The other designs presented here determine sample size and rejection regions, while controlling for both $\alpha$ and $\beta$ error rates. These designs do not consider the problem of estimation.

### *Fleming's designs*

Fleming (1982) developed designs with two or three stages. These designs allow an early termination of the trial when the intermediate results are extreme, either in favour of the efficacy or of the inefficacy of the treatment. In a first step, the total number of patients is estimated as if one was planning a single-stage trial with error rates $\alpha$ and $\beta$ for success rates respectively larger than $p_1$ and smaller than $p_0$. The number of stages is then selected arbitrarily, usually between two and three, and the total number of patients is divided arbitrarily, in general equally, between the stages. In the last step, the cut-off points are defined by the required values for $\alpha$ and $\beta$.

### *Simon's designs*

Simon (1989) proposed two designs, each with two stages. Simon's Optimum design minimises the average number of patients exposed to an ineffective treatment and his Minimax design minimises the maximum sample size required. In these designs, the number of patients in each stage is not specified by the investigator, unlike Fleming's design, but is a result of the minimisation constraint.

In Simon's original proposal, early termination of the trial was considered possible only when the results supported the hypothesis of treatment inefficacy. The calculations presented here use a modification of Simon's designs where one stops the trial at the end of the first stage if the accumulated number of successes reaches the upper cut-off point of the second stage. A conclusion in favour of efficacy is then reached at the end of the first stage.

### *Ensign's design*

Ensign *et al.* (1994) proposed a three-stage design which combines Gehan's first stage with Simon's Optimum two-stage design. It allows early termination of a trial beginning with a long run of failures. The first stage is thus constructed as Gehan's first stage: $n_1$ patients are treated and if no successes are observed, the trial is stopped. If at least one success is observed among the first $n_1$ patients, then the two-stage Simon's Optimum design is used.

### Comparision of designs

We have compared the designs, using typical error rates of $\alpha = 0.05$ and $\beta = 0.10$. The treatment was considered as not sufficiently effective if the true proportion of successes was less than or equal to $p_0 = 10\%$. If the true proportion of successes was greater than or equal to $p_1 = 30\%$, the treatment was considered as worthy of study in a phase III trial. These proportions, $p_0$ and $p_1$ are symmetrical around 20%, a success rate frequently used in phase II oncology trials which study one treatment.

In principle, Gehan's design cannot be compared with the other designs since it specifies the error rate $\beta_1$ but does not specify the error rate $\alpha$. In order to guarantee an overall $\beta$ error rate of 10%, we have chosen a design which satisfies Gehan's constraint of $\beta_1 = 5\%$. This leads to the inclusion of nine patients in the first step. The total number of patients equal to 35 has been selected according to Fleming's design (see below). This leads to an overall $\beta$ error of 9% for $p_1 = 30\%$.

Table I presents, for each design, the number of stages, the cut-off points and the sample size. It also includes the probabilities of early termination (PET) and the average sample sizes (AN) under the hypotheses of inefficacy ($p_0 = 10\%$) and of efficacy ($p_1 = 30\%$).

To show how Table I should be read, we consider Fleming's design. The total sample size is 35 patients, which is the number of patients required in a single-stage design with $\alpha = 5\%$ and $\beta$ below 10%. The division of the 35 patients between the two stages in arbitrary, and we have chosen the sample sizes used by Fleming (1982), i.e. 20 patients for the first stage and 15 for the second stage. At the end of the first stage, if the number of successes is less than three, the trial is stopped and the treatment is judged not sufficiently effective. If six or more successes are observed, the trial is stopped and the treatment is judged effective enough for further study in a phase III trial. If three, four or five successes are observed, 15 more patients are recruited into the study. Once these 15 patients are included, if the total number of successes observed in the 35 patients is less than seven, the treatment is concluded to be ineffective. If the number of successes is at least seven, it is concluded to be effective. With this particular design, the overall error rate $\alpha$ is equal to 0.053, which is quite close to the nominal level of 5%, and the overall error rate $\beta$ is equal to 0.080, which is

**Table I** Comparison of sample size, cut-off points, probability of early termination (PET) and average sample size (AN) for five phase II designs, with $\alpha \approx 0.05$ and $\beta \approx 0.10$

| *Design* | *Number of stages* | *Stage* k | *Sample size at stage* k | *Cumulative sample size* | *Conclude ineffectiveness if* $R_k$ | *Conclude effectiveness if* $R_k$ | $p_o = 10\%$ *PET* | $p_o = 10\%$ *AN* | $p_1 = 30\%$ *PET* | $p_1 = 30\%$ *AN* |
|---|---|---|---|---|---|---|---|---|---|---|
| Gehan | Two | | | | | | | | | |
| | | 1 | 9 | 9 | =0 | ⩾7 | 39% | | 5% | |
| | | 2 | 26 | 35 | ⩽6 | ⩾7 | | 24.9 | | 33.8 |
| Fleming | Two | | | | | | | | | |
| | | 1 | 20 | 20 | ⩽2 | ⩾6 | 69% | | 62% | |
| | | 2 | 15 | 35 | ⩽6 | ⩾7 | | 24.7 | | 25.7 |
| Simon's Optimum | Two | | | | | | | | | |
| | | 1 | 18 | 18 | ⩽2 | ⩾7 | 74% | | 34% | |
| | | 2 | 17 | 35 | ⩽6 | ⩾7 | | 22.5 | | 29.2 |
| Simon's Minimax | Two | | | | | | | | | |
| | | 1 | 22 | 22 | ⩽2 | ⩾7 | 62% | | 53% | |
| | | 2 | 11 | 33 | ⩽6 | ⩾7 | | 26.1 | | 27.2 |
| Ensign | Three | | | | | | | | | |
| | | 1 | 9 | 9 | =0 | ⩾8 | 39% | | 4% | |
| | | 2 | 13 | 22 | ⩽3 | ⩾8 | 84%[a] | | 42%[a] | |
| | | 3 | 23 | 45 | ⩽7 | ⩾8 | | 20.6 | | 34.8 |

*R* is the cumulative number of successes at the end of stage *k*. [a]Including the probability of termination at the end of stage 1.

reasonably close to 10%. If the treatment has a true efficacy of 10%, the average number of patients (AN) is 24.7 and the probability of early termination (PET) after the first stage is 69%. This PET is the sum of the probability of early termination with a correct conclusion of inefficacy (68%) and of the probability $\alpha_1$ of early termination with a wrong conclusion of efficacy (1%). If the treatment has a true efficacy of 30%, the average number of patients is 25.7 and the probability of early termination after the first stage is 62%. This PET is the sum of the probability of early termination with a correct conclusion of efficacy (58%) and of the probability $\beta_1$ of early termination with a wrong conclusion of inefficacy (4%).

Comparing the different designs, one can seen that the maximal sample size varies from 33 for Simon's Minimax design to 45 patients for Ensign's design. The number of patients included in the first stage varies from nine for Gehan's and Ensign's designs to 22 for Simon's Minimax design.

After the first stage, Ensign's design, like Gehan's design, always stops with a conclusion of inefficacy if no successes are observed; the other designs stop with a conclusion of inefficacy if fewer than three successes are observed.

Under the hypothesis of inefficacy, Ensign's design and Simon's Optimum design have the largest overall probabilities of early termination of the trial (84% and 74%). Among the designs presented in Table I, Gehan's and Ensign's design have the smallest probability of early termination at the end of the first stage. In general, a larger probability of early termination corresponds to a smaller average sample size.

Under the hypothesis of efficacy, Fleming's design leads to the highest probability of early termination and to the smallest average sample size followed by Simon's Minimax design, Simon's Optimum design, Gehan's design and Ensign's design.

We have studied the robustness of the ranking of the five designs to variations of $\alpha$, $\beta$, $p_0$ and $p_1$, considering all nine combinations of the selection of either $\alpha=5\%$, $\beta=10\%$, or $\alpha=5\%$, $\beta=5\%$, or $\alpha=10\%$, $\beta=10\%$, and the selection of either $p_0=10\%$, $p_1=30\%$, or $p_0=20\%$, $p_1=40\%$, or $p_0=30\%$, $p_1=50\%$. In general, under the hypothesis of inefficacy, Ensign's design had the largest overall probability of early termination, followed by Simon's Optimum design, Fleming's design and Simon's Minimax design; Gehan's design having the smallest probability of early termination. Under the hypothesis of efficacy, the largest probability of early termination was obtained with Fleming's design, followed by Ensign's design, Simon's Minimum and Simon's Optimum designs; Gehan's design corresponding to the smallest probability of early termination.

### Literature review

We have analysed 3–10 issues published between January and November 1995 of five journals: *American Journal of Clinical Oncology, British Journal of Cancer, Cancer, European Journal of Cancer* and *Journal of Clinical Oncology*, searching for chemotherapy trials in oncology that were either described as 'phase II' anywhere in the text, or had tumour response for main end point (these trials being sometimes described as 'pilot studies').

A total of 83 papers reporting phase II studies as defined above, were identified: 26 in the *American Journal of Clinical Oncology*, 18 in the *European Journal of Cancer*, 14 in the *Journal of Clinical Oncology*, 13 in *Cancer* and 12 in the *British Journal of Cancer*.

Out of these 83 papers, ten (12%) included at least some information on the statistical technique used: four reported using Gehan's design, one Fleming's design, two Simon's design, one an 'optimal restricted Bayes sampling', the last two specified $p_0$ and the planned total number of patients, without specifying the number of stages, one specifying also the values of $\alpha$, $\beta$, and $p_1$. The other 73 papers did not specify any of the following: name of statistical method, number of stages, $\alpha$, $\beta$, $p_0$ and $p_1$. Among these 73 trials, the 11 in which zero response has been observed could be considered as having been conducted according to Gehan's design. With this extremely optimistic interpretation, the proportion of trials having used an identifiable statistical design rises to 25%.

### Discussion

Gehan's design is simple and easy to understand. It is quite satisfactory for trials that are not to be stopped when the treatment is very effective. However, it tends to expose more patients to an ineffective treatment, which may raise ethical questions, particularly when treating a serious illness. Gehan's method was published more than 30 years ago and is still widely used with many phase II studies including 14 patients in the first stage. Nevertheless, the second stage is rarely carried out, even when successes are observed during the first stage. This may be because the sample sizes suggested by Gehan's tables for the second stage are often too large for phase II trials.

Fleming's design is recommended when one wants to terminate the trial as soon as the effectiveness is considered to have been demonstrated. It has the highest probability of early termination under the efficacy hypothesis. In Fleming's design, the total number of patients is estimated as if one was planning a single-stage trial with the selected error rates. Sample sizes for each stage are then selected arbitrarily, and the decision-making rules are defined by the required values for $\alpha$ and $\beta$. In Simon's and Ensign's designs, on the other hand, the sample size is not defined by the investigator, but depends on the minimisation criterion.

In Simon's Optimum design, under the hypothesis of treatment inefficacy, the probability of early termination of the trial is large, and the average number of patients is minimum.

In the case of an extremely rare disease and thus a low accrual rate, Simon's Minimax design will be preferred to the Optimum design since it limits the maximum duration of the study. For instance, with $p_0=0.10$, $p_1=0.30$, $\alpha=0.10$ and $\beta=0.10$, Simon's Minimax design leads to a maximum of 25 patients under the efficacy hypothesis. Under the same hypothesis, Simon's Optimum design leads to a maximum of 35 patients. If the accrual rate is ten patients per year, this difference could represent one extra year of accrual compared with Simon's Minimax design.

One of the drawbacks of the two designs proposed by Simon is that they do not allow the trial to be stopped early when there is a long series of failures at the beginning. For example, Simon's Optimum design for $p_0=0.20$, $p_1=0.35$, $\alpha=0.10$ and $\beta=0.10$ requires a sample size at the first stage of $n_1=27$ patients with early termination when five or fewer successes have been observed. This implies stopping only when at least 22 failures have been observed, even if the first 21 patients fail. Most investigators would want to stop a trial with such poor results earlier, and this is the argument underlying Ensign's design.

If it is likely that the treatment is ineffective, which may often be the case in phase II trials in oncology, Ensign's design is recommended since it allows for premature stopping of the trial at the end of the first stage if no successes have been observed among the first $n_1$ patients, while preserving the nominal $\alpha$ and $\beta$ errors.

When selecting the error probabilities, $\alpha$ and $\beta$, the investigator should be aware of their interpretations. The type I error, $\alpha$, is the probability of concluding wrongly that the efficacy of a treatment justifies the undertaking of a phase III trial. This error will lead to treating additional patients with an ineffective treatment, and wasting addition funds. To limit these risks, one would select a small value for $\alpha$. The type II error, $\beta$, is the probability of concluding that the treatment is inefficient when in fact it is efficient. The plan to undertake a phase III trial would then be abandoned in this case. This error saves money but misses the chance to study a potentially useful treatment. It is often considered as less important an error than $\alpha$. Should this not be the case, one can select a small value for $\beta$. In general the design with more patients during the first stage is preferable since the corresponding $\beta$ error rate is small. The probability of falsely concluding the treatment to be ineffective at the end of the first stage will therefore be low.

It should be noted that phase II trials in cancer use response as the outcome, whereas phase III trials use time to recurrence or time to death. There may not be a strong association between tumour response and patient survival. Phase III trials are therefore essential for studying the potential benefit in terms of survival and disease-free survival.

Many trial protocols and most published reports of phase II studies do not mention the design used. Statistical considerations should be part of every phase II trial protocol. These statistical considerations should include a short description of the design with a reference, a justification of the choice of design, and the values of the parameters selected. The report of a phase II study should include this information, but it should also present a description of the type and duration of the responses, and a description of toxicity. All these elements will contribute to the decision to undertake a phase III trial.

### Bullet points

The protocol of a phase II trial should include statistical considerations including:

(1) a definition of the end points defining success;
(2) the name of the design used and a reference to a published description of the method;
(3) the success rate $p_0$ below which one wants to reject the treatment;
(4) $\alpha$, the probability of a conclusion in favour of efficacy when the rate of success is $p_0$ or less;
(5) The success rate $p_1$ above which one wants to accept the treatment;
(6) $\beta$, the probability of a conclusion of inefficacy when the level of efficacy is above $p_1$.

Publications reporting the results of a phase II trial should include the same information. It should also present the observed success rate with its confidence interval.

**Acknowledgements**
We thank Laurent Vabre for his assistance with the literature review, and the referees for their constructive comments.

### References

ENSIGN LG, GEHAN EA, KAMEN DS AND THALL P. (1994). An optimal three-stage design for phase II clinical trials. *Stat. Med.*, **13**, 1727–1736.

FLEMING TR. (1982). One sample multiple testing procedure for phase II clinical trials. *Biometrics*, **38**, 143–151.

GEHAN EA. (1961). The determination of the number of patients required in a preliminary and a follow-up trial of a new chemotherapy agent. *J. Chron. Dis.*, **13**, 346–353.

MACHIN D, CAMPBELL MJ, FAYERS PM AND PINOL A. (1996). Sample size tables for clinical studies. Blackwell Scientific Publications: Oxford.

SIMON R. (1989). Optimal two-stage designs for phase II clinical trials. *Controlled Clin. Trials*, **10**, 1–10.


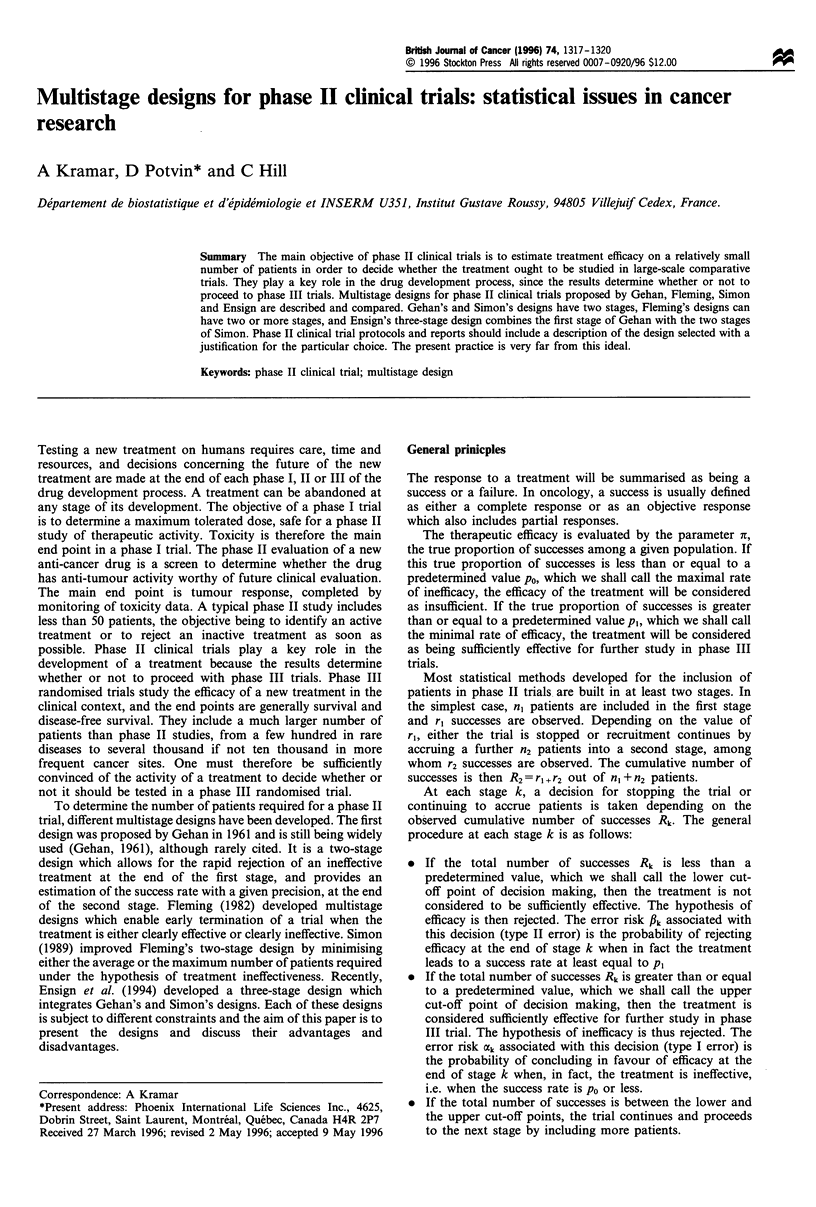

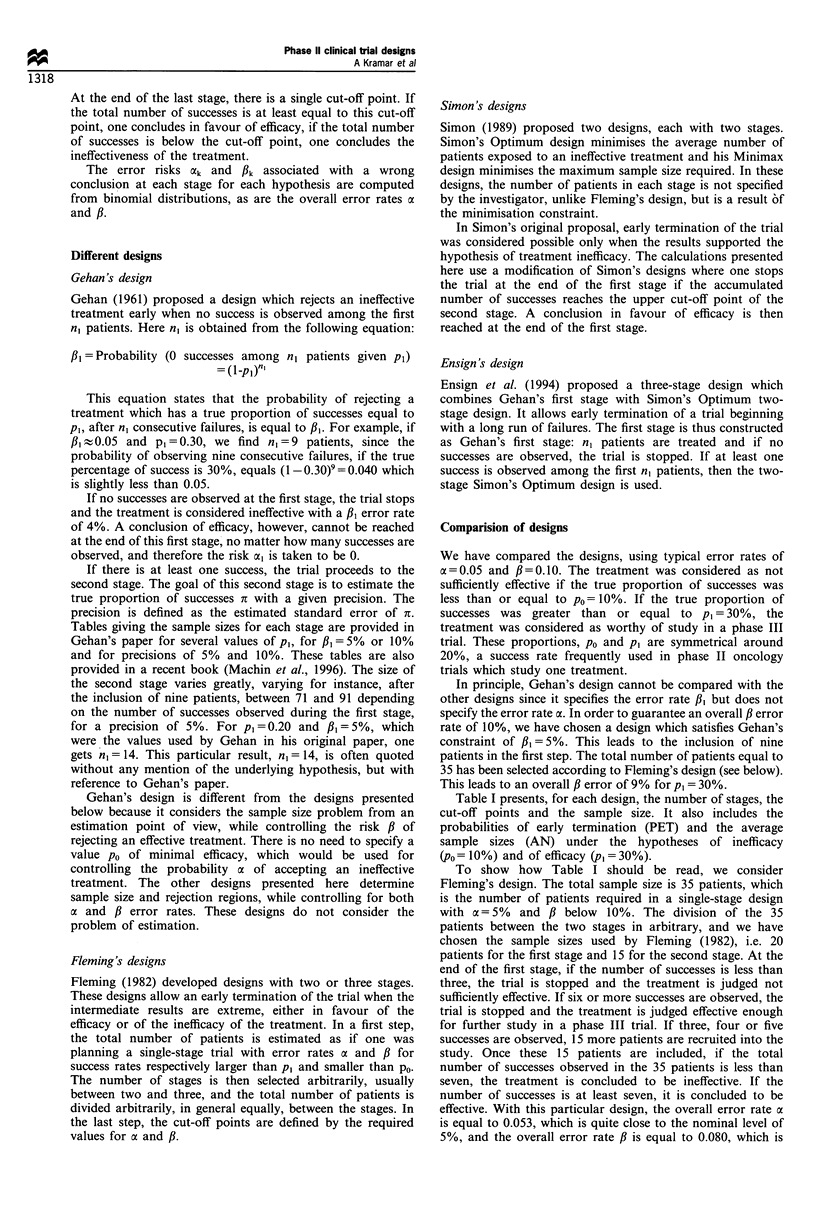

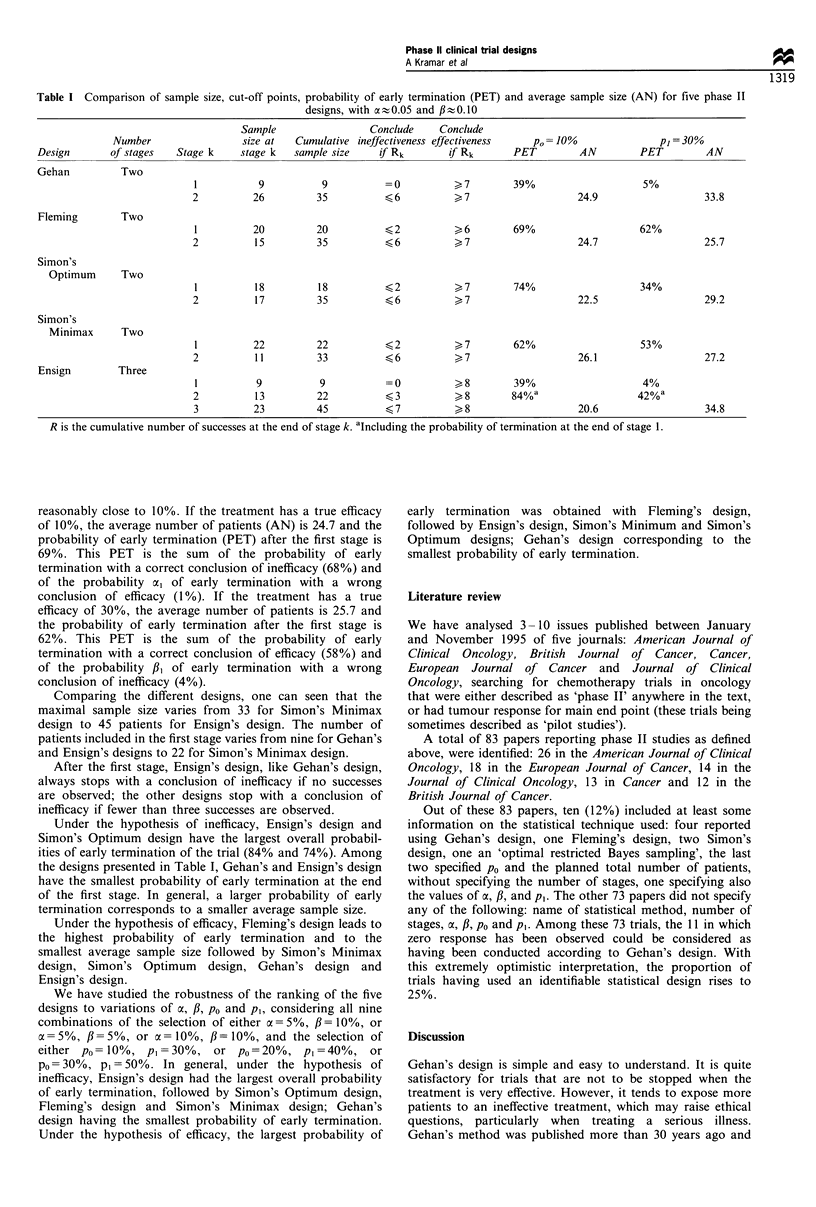

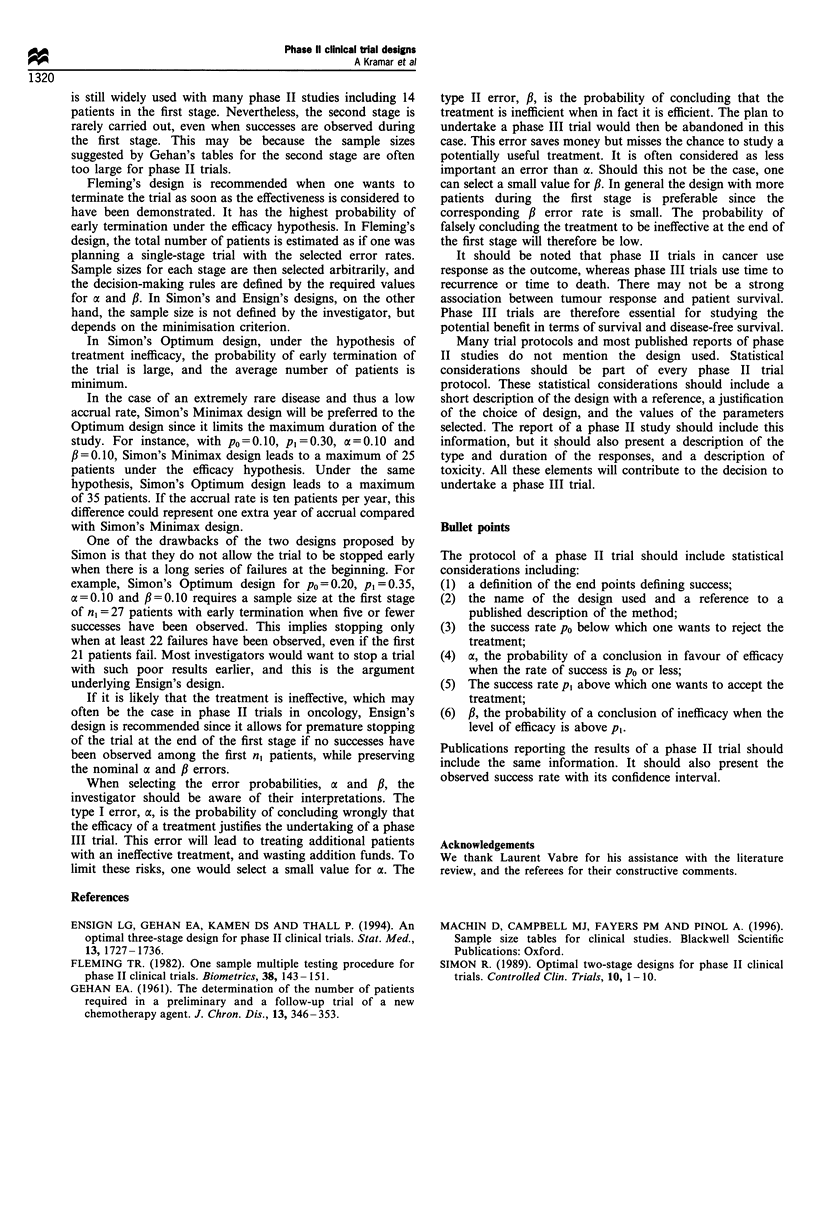


## References

[OCR_00663] Ensign L. G., Gehan E. A., Kamen D. S., Thall P. F. (1994). An optimal three-stage design for phase II clinical trials.. Stat Med.

[OCR_00668] Fleming T. R. (1982). One-sample multiple testing procedure for phase II clinical trials.. Biometrics.

[OCR_00672] GEHAN E. A. (1961). The determinatio of the number of patients required in a preliminary and a follow-up trial of a new chemotherapeutic agent.. J Chronic Dis.

[OCR_00682] Simon R. (1989). Optimal two-stage designs for phase II clinical trials.. Control Clin Trials.

